# Association of two common polymorphisms of apolipoprotein A5 gene with metabolic syndrome indicators in a North Iranian population, a cross-sectional study

**DOI:** 10.1186/2251-6581-13-48

**Published:** 2014-04-07

**Authors:** Sohrab Halalkhor, Farzad Jalali, Karimollah Hajian Tilaki, Shahla Shojaei

**Affiliations:** 1 Department of Biochemistry and Biophysics, School of Medicine, Babol University of Medical Sciences, Babol, Iran; 2 Department of Cardiology, School of Medicine, Babol University of Medical Sciences, Babol, Iran; 3 Department of Social Medicine, School of Medicine, Babol University of Medical Sciences, Babol, Iran; 4 Department of Biochemistry, School of Medicine, Shiraz University of Medical Sciences, Shiraz, Iran

**Keywords:** -1131T>C (rs662799), c.56C>G (S19W, rs3135506), Triglyceride, WHR

## Abstract

**Background:**

Metabolic syndrome is an obesity dependent disorder with a worldwide high prevalence. Regarding the high prevalence of Metabolic syndrome in Iran we analyzed the influence of -1131T>C (rs662799) and c.56C>G (S19W, rs3135506) polymorphisms of the novel apolipoprotein gene, ApoA5, on some Metabolic Syndrome indicators in population from north of Iran.

**Methods:**

199 volunteers from Babol city-Iran were divided in two groups of low (N = 99, TG ≤ 103 mg/dl) and high (N = 100, TG ≥ 150 mg/dl) serum levels of Triglycerides (TG). We amplified the gene fragments containing -1131T>C and c.56C>G polymorphisms by PCR method and revealed the polymorphisms by RFLP analysis.

**Results:**

We found a significant association (p = 0.016, Independent t-test) between high levels of TG and -1131T>C polymorphism but not between this polymorphism and serum HDL-C concentrations. Carriers of the C allele had a 1.97 times higher odds ratio to be in the high-TG group than those of the TT genotype (95%, CI = 1.05-3.68). We observed no association between -1131T>C polymorphism with either Waist-to-Hip Ratio (WHR) or Body-Mass-Index (BMI). In the case of c.56C>G polymorphism, although it showed a significant relationship with WHR (p = 0/040, Independent t-test), but failed to correlate with either levels of TG (p = 0.594) or HDL-C (p = 0.640) in serum.

**Conclusion:**

Our study confirms that ApoA5 gene polymorphisms, -1131T>C and c.56C>G are associated with the two criteria of Metabolic Syndrome, TG and WHR, respectively.

## Background

Metabolic syndrome (MetS) is a combination of clinical and paraclinical signs characterized by abdominal obesity, dyslipidemia (high triglyceride (TG), low HDL-cholesterol (HDL-C)), hypertension and hyperglycemia (3 out of 5). Several definitions have been presented to diagnose MetS, with the most earliest being the WHO definition, which declares high TG and waist-to-hip ratio (WHR) as the basic criteria [1]. Recent studies presented hypertriglyceridemia as a hallmark of MetS [2]. Also, Hypertriglyceridemia can subsequently result in serum HDL-C reduction, another hallmark of MetS [2,3].

MetS has been known as a risk factor for cardiovascular disease (CVD) and diabetes mellitus [4] and has an increasing rate of prevalence globally [5,6]. The prevalence of MetS based on National Cholesterol Education Program Adult Treatment Panel III (NCEP ATP III) in Iran varies from 21% in southeast [7] to 23.7% in the west [8] and 29.9% in the center [9] with higher rate in women than men. It is worth mentioning that the prevalence of MetS is 31% among women in the north [10].

The exact pathological mechanism of MetS has not yet been clarified but it has been established that both environmental and genetic factors are involve [11-13]. ApolipoproteinA5 (ApoA5) gene which is located on chromosome 11q23 (NC_000011.9) on APOA1/C3/A4/A5 gene cluster has been recently discovered [14]. Despite its low plasma concentration, ApoA5 has been shown to have great effect on the plasma TG concentration [15,16]. These effects are partially attributed to the role of ApoA5 on TG metabolism (Reviewed in [17]) as well as to its influence on food intake [18].

Molecular mechanism of ApoA5 effect on TG metabolism is not completely understood. ApoA5 is suggested to impede the second step of VLDL gathering [19]. In vivo studies on human ApoA5 transgenic mice demonstrated that ApoA5 could interact with lipoprotein lipase and significantly increase its activity [20]. These results suggest that ApoA5 might induce a decrease in VLDL associated TG levels by both decreasing hepatic VLDL synthesis and increasing VLDL clearance. Besides, a recent study showed that ApoA5 may be absorbed by human adipocytes and may play a role in TG storage regulation [21].

Two polymorphisms of ApoA5, -1131T>C (rs662799) and c.56C>G (rs3135506) have been shown to be associated with TG level and dyslipidemia in different ethnics [22-25]. ApoA5 -1131T>C polymorphism is located in the promoter of ApoA5 gene; therefore, it is expected to affect gene transcription and consequently serum ApoA5 levels. Relationship between ApoA5 level and TG has been reported in some research [26].

c.56C>G polymorphism of APOA5 gene has been identified as a functional variant which tryptophan is substituted with serine in signal peptide. This polymorphism results in decrease in ApoA5 secretion and 3 fold decline in plasma protein level with subsequent increase in plasma TG [27].

With the aim of addressing the role of APOA5 polymorphisms in the high prevalence of MetS in the north of Iran, we investigated the association of the two common polymorphisms of ApoA5 gene, -1131T>C and c.56C>G, with the MetS criteria.

### Subjects, material and methods

#### Ethical consideration

This cross-sectional study was performed from August 2008 to August 2009 in Babol, Iran. This study was done in agreement with the Helsinki Declaration, following approval by the ethical committee of the Medical school. Written informed permission was obtained from all subjects after ensuring them about safety of the procedure and security of their information.

#### Subjects

The study subjects were 199 men and woman with the age of 30 to 73 years old. Volunteers had referred to Pars laboratory in Babol-Iran from 2008-08-01 to 2009-08-17. We obtained written, informed consent from the subjects. Their personal data including age, living place, cigarette and alcohol consumption, past medical history and drugs consumption were collected through an interview. Those who had diabetes, any thyroid disorders or being treated for these disorders or who had an abnormal FBS or TSH serum levels were excluded. Everyone with serum cholesterol more than 300 mg/dl or treating with any drugs that influence on lipid metabolism was omitted. Besides, people with alcohol consumption or severe weight loss during the previous two weeks were excluded too. Anthropometric characteristics of the included individuals such as: height, weight, hip and waist circumferences were measured by a trained person, and then BMI and WHR were calculated. The subjects based on serum TG level were divided into two groups: low TG (TG ≤ 103 mg/dl) including 49 men and 50 women and high TG (TG ≥ 150 mg/dl) including 50 men and 50 women.

#### Biochemical and DNA Analyses

10 ml of blood sample was taken from each individual after overnight fasting, 5 ml for biochemical and 5 ml for DNA extraction. Serum was separated immediately after clotting and serum FBS, TG, total cholesterol, HDL-C and LDL-cholesterol (LDL-C) levels were measured by BS-300 MINDRAY (Shenzhen Mindray Bio-medical Electronics Co., China) by DIASIS kits (Germany). Serum TSH was measured by (AWARENESE, Stat Fax-200 model), using a commercially available ELISA DIAPLUS, INC (Q1) kits (America). For quality control purpose TruCal U Lot: 10929 and TruCal HDL/LDL, Lot: 10502 were used to calibrate the biochemical tests and TruLab N, P Lot: 11382 and TruLab Lipid, Lot: 10501 were applied to check the accuracy of biochemical tests. To control the quality of TSH tests Dia Plus EIA, RIA, CLIA Control Serum, Lot: MC1A5 was used.

Genomic DNA was extracted by the salting out method from the peripheral blood. Genes of interest were amplified by 200 mM dNTP, 2U SmarTaq (Cinagen, Iran), 1× smarTaq buffer, 200 nM each primer 1.5 mM MgCl_2_ and 1 ul of DNA were mixed and the volume was adjusted to 25 ul with water. PCR was performed by PRIMUS (MWG-Biotech) thermal cycler. -1131T>C polymorphism was amplified at 95°C for 12 min followed by 30 cycles of 94°C for 15 s, 55°C for 30s, and 72°C for 30s and a final extension at 72°C for 3 min to produce 187 bp fragments. Products were digested with MseI (Tru1I) and resolved on 3% agarose gels post-stained with ethidium bromide and imaged with SYNGENE transilluminator and INGENIUS (SYNGENE BIO IMAGING) Gel Duct system. The resulted segments in the presence of common allele T were 167 and 20 bp versus non digested 187 bp segment in the presence of rare allele C.

For c.56C>G polymorphism fragment of interest was amplified at 95°C for 10 min followed by 35 cycles of 95°C for 35 s, 58°C for 25 s and 72°C for 30s and a final extension at 72°C for 2 min to produce 292 bp fragment. PCR products were digested with Sau96I (Cfr13I) enzyme. After digestion we had 210 and 82 bp segments in the presence of common allele C and 150, 82 and 60 bp segments in the presence of rare allele G. Genotypes were determined two times using blind reader method. Sequences of used primer for each polymorphism have been demonstrated in Table 1.

**Table 1:** Sequences of primers used for each polymorphism

**Polymorphism**	**Forward primer (5′ > 3′)**	**Reverse primer (5′ > 3′)**
-1131T>C (rs662799) [34]	GATTGATTCAAGATGCATTTAGGAC	CCCCAGGAACTGGAGCGAAATT
c.56C>G (rs3135506) [23]	CCACCCTGGGGGAGGAGAGCCCAAGCCCTG	AGAGCTAGCACCGCTCCTT

#### Statistical analyses

Statistical analyses were done using SPSS 10 and Microsoft excel. Allele frequencies in low and high TG groups were accessed by χ2 test. Associations between genotypes, lipids, and anthropometric parameters were evaluated using independent t-test and One-way ANOVA. Lipid levels were expressed in mg/dl and all values were reported as means ± SD. P < 0.05 (two-tailed) was considered significant. Odds ratio was analyzed through logistic regression.

## Results

**Figure 1:**
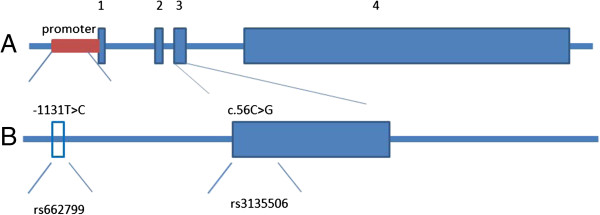
**Maps of the human APOA5 gene with polymorphism positions indicated. (A)** Map of the human APOA5 gene. Exons 1-4 are numbered and represented by solid boxes. **(B)** Position of the single nucleotide polymorphism within the core promoter of the APOA5 gene, a T/C polymorphism at position -1131 and a C/G polymorphism at position 56.

The groups were included 99 low TG (50 male age: 43.30 ± 12.15 and 49 female age: 46.53 ± 10.90 years) and 100 high TG (50 male age; 45.78 ± 11.20 and 50 female 48.40 ± 9.25 years) volunteers. The human APOA5 gene map with polymorphism positions indicated in Figure 1.

### -1131T>C polymorphism

Allele frequencies of this polymorphism were in Hardy–Weinberg equilibrium in both groups of high and low TG. Genotypes in the high (TG ≥150 mg/dl) and low (TG ≤103 mg/dl) TG groups have been shown in Table 2. Both CC and TC genotypes were significantly associated with the high TG group (P = 0.02, χ2 test). The frequencies of the rare allele C in the high and low TG groups were 0.21 and 0.11 respectively. We found a higher number of CC homozygotes in the high TG group as compared with the low TG group. Likewise, the number of TC heterozygotes in the high TG group was higher than those in the low TG group.

**Table 2:** Allele frequency and genotype percent of APOA5 -1131T>C (rs662799) in association with TG

**Genotype**	**TG**	
	**High**^**1**^	**Low**^**2**^
TT	64 (64%)	77 (77.8%)
TC	31 (31%)	22 (22.2%)
CC	5 (5%)	0 (0%)
Total	100	99

1: High TG (≥150 mg/dl), 2: Low TG (≤ 103 mg/dl).

We also considered CC and TC genotypes in one group (allele C carriers) and used independent t-test to compare the biochemical and anthropometric parameters of subjects in this group with those of the volunteers with the TT genotype. Thus, a significant mean difference was observed only between the levels of TG in the two above mentioned groups (Table 3).

**Table 3:** Mean ± SD and P-value significance for phenotype analysis of -1131T>C (rs662799) polymorphism

**Parameter**	**Genotype**		**P-value**^**£**^
	**TT**	**CC and TC**	
TG (mg/dl)	153.46 ± 87.81	196.69 ± 154.36	0.016
Cholesterol (mg/dl)	196.48 ± 39.98	200.12 ± 41.67	0.565
LDL-C (mg/dl)	119.46 ± 31.73	116.48 ± 28.31	0.537
HDL-C (mg/dl)	47.19 ± 13.19	44.74 ± 14.20	0.247
BMI (kg/m^2^)	28.50 ± 4.68	29.41 ± 6.16	0.260
WHR	0.90 ± 0.08	0.89 ± 0.07	0.229

£: Independent t-test.

When the subjects were classified according to sex, differences between the TG levels of men with the TT genotype and those with CC and TC genotypes remained statistically significant (Table 4). This difference did not achieve significance with respect to women.

**Table 4:** Mean ± SD for biochemical and anthropometric analysis of -1131T>C (rs662799) polymorphism and P-value of significance for CC/TC versus TT genotype in men and women

**Parameter**	**Women**			**Men**		
	**Genotype**		**P-value**^**£**^	**Genotype**		**P-value**^**£**^
	**CC & TC**	**TT**		**CC & TC**	**TT**	
TG (mg/dl)	178.04 ± 90.89	156.63 ± 95/09	0.310	212.17 ± 196.36	150.24 ± 80.30	0.027
Cholesterol (mg/dl)	193.43 ± 35.43	189.30 ± 39.86	0.633	206.37 ± 46.48	203.77 ± 39.04	0.774
HDL-C (mg/dl)	41.82 ± 13.59	43.19 ± 11.49	0.616	47.47 ± 14.44	51.19 ± 13.64	0.223
BMI (Kg/m^2^)	27.45 ± 3.68	27.63 ± 4.39	0.845	31.24 ± 7.41	29.37 ± 4.83	0.137
WHR	0.93 ± 0.06	0.92 ± 0.07	0.869	0.85 ± 0.06	0.89 ± 0.09	0.092

£: Independent t-test.

Results of logistic regression analysis also showed that the allele C carriers were associated with a high TG with an odds ratio of 1.97 (95%, CI = 1.05-3.68) when compared with subjects with TT genotype (Table 5). When odds ratio was adjusted with BMI, it remained two fold but not significant yet.

**Table 5:** Odds ratio for the allele C carriers versus TT genotypes of -1131T>C (rs662799) polymorphism in occurrence of high TG

	**Unadjusted model**		**Adjusted with BMI**	
	**OR (CI 95%)**	**P-value**^**£**^	**OR (CI 95%)**	**P-value**^**£**^
Allele C carriers versus TT genotype	1.97 (1.05–3.68)	0.034	2.03 (0.89–4.60)	0.090

£: logistic regression.

### c.56C>G polymorphism

The frequencies of allele G in the two groups of high and low TG were respectively 0.055 and 0.045 that were not significantly different from one another. Allele G frequency in combined high and low TG groups was 0.05 with the genotype distribution being within Hardy-Weinberg equilibrium. We found a significant association between allele G frequency and WHR but we failed to find any significant association between serum TG (P = 0.594) and HDL-C (P = 0.640) with c.56C>G polymorphism (Table 6).

**Table 6:** Mean ± SD and P-value for biochemical and anthropometric analysis of c.56C>G (rs3135506) genotype and P-value

**Parameter**	**Genotype**		**P-value**^**£**^
	**CG**	**CC**	
TG (mg/dl)	178.05 ± 98.26	163.96 ± 113.40	0.594
Cholesterol (mg/dl)	199.95 ± 36.56	197.28 ± 39.45	0.773
LDL-C (mg/dl)	122.40 ± 28.15	118.26 ± 30.19	0.559
HDL-C (mg/dl)	45.10 ± 11.85	46.61 ± 13.57	0.633
BMI (Kg/m^2^)	28.60 ± 5.84	28.86 ± 5.14	0.838
WHR	0.85 ± 0.06	0.90 ± 0.08	0.040

£: Independent t-test.

## Discussion

The results of our cross-sectional study show that ApoA5 -1131T>C polymorphism is associated with TG levels in a cohort of subjects from Babol-Iran. These data add to those of studies performed on other human populations [28-30]. Our finding that allele C carriers had a higher chance (two folds) of having a high TG than people with TT genotype is in agreement with haplotype analyses conducted on Taiwanese population [31].

Allele C frequency of this polymorphism was 21% in the high TG population (TG ≥150 mg/dl), 11% in the low TG population (TG ≤103 mg/dl) and 16% in total population. These values are close to allele C frequency reported for other Caucasian populations, such as the Brazilians 16% [32] Hispanics 13-16% and Turks 13% [23] but differ from other ethnic population like East Asians 27-37% [29,33].

Subgroup analysis revealed a significant association between the rare allele frequency and high TG just in men but not in women. These results are consistent with that reported for a Chinese population [34].

Although other investigators have reported a significant positive relationship between serum levels of total cholesterol and LDL-C with -1131T>C genotype [35], but our data failed to show such an association. The present data indicate that subjects with the TT genotype tended to show higher mean and minimum levels total cholesterol in their sera as compared to those with CC genotype. This trend was not statistically significant, but regarding the role of total cholesterol and LDL-C levels in the occurrence of CVD, it is worthy to examine if the trend will achieve significance with a larger number of volunteers.

Hypertriglyceridemia is a hallmark of MetS [2]. Regarding the great impact of -1131T>C polymorphism on the TG level, the association of this polymorphism with MetS has been the subject of many population genetic studies. Thus, the association of -1131T>C polymorphism with MetS has been shown in Korean [36] and Chinese populations [37]. A meta-analysis also showed the significant association of this polymorphism with MetS in Asian, but not in white people [38].

Association of this allele with a spectrum of cardiac diseases has been shown. Lima et al. reported this association with atheromatosis in Coronary Artery Disease [39] and Ding et al. with Acute Coronary Syndrome [40]. Besides, a meta-analysis has indicated the association of this variant with risk of Ischemic Stroke [41]. Also, the association of -1131T>C polymorphism with CVD has been shown in studies done on various populations, including Chinese [42], Indian [43] and Korean [44].

With respect to the location of -1131T>C polymorphism in the promoter of ApoA5 gene, its effect on gene transcription and consequently serum ApoA5 levels sounds reasonable. It seems that presence of allele C, by reducing ApoA5 level, results in an increase in TG level [44]. Although, relationship between this variant with ApoA5 level and TG has been reported in some studies [26], this association was independent of ApoA5 levels in some others [45]. Therefore, it has been hypothesized that this association might be because of the strong relationship between -1131T>C polymorphism with disequilibrium of polymorphisms of ApoC3 gene [46].

The frequency of the G Allele of ApoA5 c.56C>G polymorphism in our population (5%) is close to the corresponding values reported for Caucasians such as Danish 6% [24], Turks 6% [47] and British 6% [48], as well as populations like Brazilians 7% [32], but it is less than that obtained for Costaricans 10.2% [49]. The reason for similarities between results we obtained for our Iranian population and those obtained for Caucasians can be attributed to the close ethnic relationship between the two populations. Despite the low frequency of the G allele of the c.56C>G polymorphism in our population, we detected a significant association between this minor allele with WHR, a finding that points to the strong influence of this polymorphism on anthropometric parameters. The interaction of G Allele of ApoA5 c.56C>G polymorphism with LPL polymorphism to heighten the genetic susceptibility to obesity have also been reported [50]. However, to our knowledge, there is no data on the independent effect of ApoA5 c.56C>G polymorphism on the anthropometric parameters in the literature.

WHR is shown to be a better predictor for MetS and some other diseases [51-53]. Our present data indicate that G allele of ApoA5 c.56C>G polymorphism had a great impact on WHR. Therefore it is of interest to investigate other ethnic groups to confirm the influence of this ApoA5 polymorphism on the WHR.

Results of a number of studies have shown an association between the G allele of ApoA5 c.56C>G polymorphism and levels of TG, both in the general population [45,54] and in subgroups of patients, like diabetics [55] and type III hyperlipidemias [56]. Although, the mean TG level in our subjects who were heterozygotes for allele G was higher than that of non-carriers but possibly due to the small sample size, the difference was not statistically significant. However, in consistent with our data, a study on 1703 Costarican subjects didn’t find any association between this variant and TG level [49]. Likewise, data of another study on 1020 Puerto Rican subjects, showed that c.56C>G polymorphism was associated with levels of HDL-C but not TG in the sera of their subjects [57]. These inconsistencies may be due to ethnic differences or linkage disequilibrium with other genetic factors.

Association of the minor allele of c.56C>G polymorphism of ApoA5 with decreased HDL-C [25,49,57] and increased LDL-C and total cholesterol [47] has been reported. However, our data didn’t show this association, maybe because of aforementioned reason.

In the end it is very important to consider the strengths and weakness of the current study. One of the highlights of our study is age and gender matched groups, which significantly decrease the effects of these factors on the outcomes of the study. On the other hand, there are some limitation in this work including low sample size, especially for c.56C>G polymorphism that have lower frequency. Finally, we couldn’t evaluate the association of all MetS indicators with these polymorphisms that would be the target of our future studies.

## Conclusion

Our results indicate the association of two common polymorphism of ApoA5 gene, -1131T>C and c.56C>G, with respectively TG and WHR, both of which are indicators of MetS. It could be concluded that modification of dietary intake together with losing weight, increase the physical activity and decrease environmental stress are essential factors that might decrease MetS frequency.

## Abbreviations

ApoA5: Apolipoprotein A5; BMI: Body-Mass-Index; CVD: Cardiovascular Disease; HDL-C: HDL-Cholesterol; LDL-C: LDL-Cholesterol; MetS: Metabolic syndrome; TG: Triglycerides; WHR: Waist-to-Hip Ratio.

## Competing interests

The authors declare that they have no competing interests.

## Authors’ contributions

Prepared figure panels explaining the methodology: SS. Conceived and designed the study: SH SS. Performed the experiments: SS. Analyzed the data: SH SS KH. Contributed samples/reagents/materials/analysis tools: SH FJ SS. Wrote the paper: SH SS. Read and approved the final manuscript SH FJ KH SS.

## Authors’ information

SH Department of Biochemistry and Biophysics, FJ Department of Cardiology, KH Department of Social Medicine, School of Medicine, Babol University of Medical Sciences, Babol, Iran. SS Department of Biochemistry, School of Medicine, Shiraz University of Medical Sciences, Shiraz, Iran.
